# Supplementary material for: Benefit sharing in international rivers: A Q-methodology study of regional understanding and perception in Asia
Source: PLoS One. 2023 Jan 20;18(1):e0280625. doi: 10.1371/journal.pone.0280625 (PMC9857988; doi:10.1371/journal.pone.0280625)
Supplement: S1 File — (PDF) [file pone.0280625.s001.pdf]

Sort	No.	Statements	Sort	No.	Statements
Political /National Strategy	1	Due to the regional particularity of transboundary rivers, there is often considerable disparity between the rights and obligations for each individual riparian state.	Benefit Sharing/ Benefit Compensation(ii)	20	The benefits of water benefit sharing include indirect benefits beyond the water sector, such as promoting infrastructure construction, growth in trade, and so on.
	2	The allocation of water right is closely related to a country's national strategy.		21	Among the various benefits gained from water benefit sharing, environmental benefits to the water resources should form the basis of other types of benefits. Environmental benefits will actively promote other types of benefits, while the reverse is not necessarily true.
	3	The right to access and use of international rivers should not be referred to as water right. Instead, it should be regarded as part of the sovereignty.		22	The benefit distribution of the water benefit sharing model could take various forms of equal distribution, proportional distribution according to the required project cost, or equitable distribution of different types of benefits.
	4	International water rights over shared transboundary rivers are specific sharing schemes negotiated among riparian states, rather than a certain kind of right or interest that is well-documented, regulated, and predictable.		23	Although the principle of benefit sharing does not violate any existing principle of current international water law, it has not become an explicit obligation of any party.
	5	Among the conflicts that arose from countries that share international rivers, the more riparian states there are, the greater the differences of interests.		24	In the context where the principle of good-faith cooperation in international water law applies, countries should at least seriously consider benefit-sharing arrangements proposed by other countries.
	6	Among the conflicts that arose from countries that share international rivers, the greater the differences of national conditions (e.g. socio-economics) there are, the more claims for interests there will be.		25	The idea of water benefit sharing includes the calculation of various aspects and the consideration of relevant benefits. These include for instance, water management in industry, agriculture, domestic uses and ecological protection, which could be understood by the development of a thorough and operable index system of water benefit distribution.
	7	Among the conflicts that arose from countries that share international rivers, the more cooperation mechanisms there are, the harder it is to coordinate interests.		26	Due to the lack of any uniform standard or understanding, the concept of water benefit sharing is often ineffective in practice, achieving little more than some hollow "win-win" rhetoric.
International Law	8	Generally speaking, the principle of equitable and reasonable utilization in the Convention on the Law of the Non-Navigational Uses of International Watercourses benefits upstream countries, while the principle of not causing significant harm benefits downstream countries.		27	In the development, use and protection of transboundary river resources, countries that utilize water resources and receive ecological benefits shall correspondingly compensate countries that protect these resources and the ecological environment.
	9	The Convention on the Law of the Non-Navigational Uses of International Watercourses fails to strictly uphold the principle of fairness, in favouring the interests in water resources of the downstream countries and imposing excessive obligations on the upstream countries.		28	In the process of sharing international rivers, ecological benefit compensation should be established as a principle to guide the development, use and protection of transboundary rivers.
	10	In several aspects, such as the obligations for notification or consultation, the Convention on the Law of the Non-Navigational Uses of International Watercourses fails to abide by the principle of reciprocity, which is a fundamental principle of international law and international relations.		29	Countries may contribute to the ecological resources of transboundary rivers by taking active measures to protect resources and the ecological environment, such as forestation and the establishment of natural conservation areas.
	11	The principle of equitable and reasonable utilization in current international water law has not progressed beyond the preliminary levels of simply outlining water quantity allocation and water use division.		30	Countries may contribute to the ecological resources of transboundary rivers by spontaneously restricting or refraining from certain activities, such as withdrawing from proposed dam construction, halting construction or expansion of industrial or mining operations, or the reduction of forest logging activities.
	12	For projects that may cause significant transboundary harm, countries that share transboundary rivers shall fulfill international obligations to avoid, contain and mitigate such harm.		31	If the beneficiary countries of ecological resources in transboundary rivers do not provide reasonable compensation to those countries making contributions, it will probably dampen the enthusiasm in protecting transboundary water resources and ecological environment and negatively impact upon the improvement of relations between riparian states.
	13	Although "benefit sharing" is frequently mentioned in current international law, this concept lacks a uniform cognition, has not been fully established, and cannot be practically implemented.		32	Ecological benefit compensation will address the shortcomings of the existent "polluter pays" principle and stimulate the protection of transboundary rivers and ecological environment.
Benefit Sharing/Benefit Compensation(i)	14	Water benefit sharing theory requires that water stakeholders to be considered as a whole, with the purpose of promoting the maximization of benefits from the use of transboundary water resources, and emphasizing that all stakeholders can obtain the best benefits.		33	Ecological benefit compensation of transboundary rivers ecology may take various forms including financial compensation, material compensation, and policy support.
	15	The core idea of water benefit sharing theory is not to allocate the actual water quantity, but to share the benefits obtained through the development and utilization of water resources.		34	Under the ecological benefit compensation principle, countries that contribute to the protection of ecological resources and the environment will be motivated by the appropriate compensation they receive, in furthering their protection activities.
	16	The ideal water benefit sharing system is a positive-sum game, rather than a zero-sum game where only the amount of water available is allocated.		35	Under the ecological benefit compensation principles, beneficiary countries that have paid compensation will further appreciate the preciousness of such resources and the ecological environment that they had to pay to preserve.
	17	The benefits of water benefit sharing include environmental benefits to the water resources, such as improvement in water quality, ecological diversity, and environmental sustainability.		36	The ecological benefit compensation principle will facilitate the elevation from individual interest among riparian states to maximizing the collective benefits across the region.
	18	The benefits of water benefit sharing include direct economic benefits obtained from the uses of water resources, such as hydropower development, agricultural irrigation, and navigation convenience.		37	Any dispute in relation to the ecological benefit compensation principle could be resolved through bilateral or multilateral discussion and negotiation, failing which there may be a judicial recourse to the International Court of Justice.

No.	Eigenvalues（特征根）	As Percentages（解释样本比例）	Cumul. Percentages（累计解释样本比例）	No.	Eigenvalues（特征根）	As Percentages（解释样本比例）	Cumul. Percentages（累计解释样本比例）
1	4.8922	13.9778	13.9778	19	0.5127	1.4647	92.7480
2	3.3701	9.6288	23.6065	20	0.5000	1.4285	94.1765
3	3.0576	8.7359	32.3424	21	0.3787	1.0819	95.2584
4	2.4212	6.9177	39.2601	22	0.3158	0.9022	96.1607
5	2.1144	6.0410	45.3011	23	0.3083	0.8807	97.0414
6	2.0278	5.7936	51.0947	24	0.2565	0.7328	97.7742
7	1.9651	5.6147	56.7094	25	0.1779	0.5082	98.2824
8	1.6398	4.6850	61.3944	26	0.1716	0.4902	98.7726
9	1.5723	4.4924	65.8867	27	0.1405	0.4015	99.1740
10	1.4271	4.0773	69.9641	28	0.1157	0.3306	99.5047
11	1.3894	3.9699	73.9339	29	0.0873	0.2494	99.7541
12	1.2519	3.5768	77.5107	30	0.0544	0.1553	99.9094
13	1.0557	3.0163	80.5270	31	0.0172	0.0492	99.9585
14	0.9340	2.6686	83.1955	32	0.0098	0.0279	99.9864
15	0.8891	2.5402	85.7357	33	0.0043	0.0123	99.9987
16	0.7799	2.2282	87.9639	34	0.0005	0.0013	100.0000
17	0.6058	1.7309	89.6948	35	-0.0000	-0.0000	100.0000
18	0.5560	1.5885	91.2832				

序号	语句内容	F1		F2		F3		F4		F5		F6		F7		备注
		得分	排序	得分	排序	得分	排序	得分	排序	得分	排序	得分	排序	得分	排序	
1	Due to the regional particularity of transboundary rivers, there is often considerable disparity between the rights and obligations for each individual riparian state.	1.92	2	-1.28	34	-0.22	22	-0.33	25	-1.12	31	-0.32	26	-1.55	36	*
2	The allocation of water right is closely related to a country's national strategy.	0.00	19	0.31	15	0.45	13	0.64	10	-1.38	34	-0.15	23	0.33	15	
3	The right to access and use of international rivers should not be referred to as water right. Instead, it should be regarded as part of the sovereignty.	-1.38	34	-1.88	37	-0.94	31	1.56	2	-2.30	37	-1.80	35	-1.35	35	*
4	International water rights over shared transboundary rivers are specific sharing schemes negotiated among riparian states, rather than a certain kind of right or interest that is well-documented, regulated, and predictable.	-0.54	25	0.07	19	-0.77	28	1.25	4	-1.31	33	-2.26	36	-0.78	30	*
5	Among the conflicts that arose from countries that share international rivers, the more riparian states there are, the greater the differences of interests.	-0.15	22	0.12	18	-1.35	34	-1.59	35	-1.40	35	-0.31	25	1.55	5	*
6	Among the conflicts that arose from countries that share international rivers, the greater the differences of national conditions (e.g. socio-economics) there are, the more claims for interests there will be.	2.07	1	-0.24	25	-1.29	33	-1.24	32	-0.22	24	0.21	19	0.78	10	*
7	Among the conflicts that arose from countries that share international rivers, the more cooperation mechanisms there are, the harder it is to coordinate interests.	-0.07	21	-1.69	35	-1.57	36	0.01	19	-0.98	30	-2.48	37	-0.33	23	
8	Generally speaking, the principle of equitable and reasonable utilization in the Convention on the Law of the Non-Navigational Uses of International Watercourses benefits upstream countries, while the principle of not causing significant harm benefits downstream countries.	0.00	19	-1.10	32	-0.52	27	0.62	11	-0.70	28	0.27	17	0.33	15	
9	The Convention on the Law of the Non-Navigational Uses of International Watercourses fails to strictly uphold the principle of fairness, in favouring the interests in water resources of the downstream countries and imposing excessive obligations on the upstream countries.	-1.23	33	-0.23	24	0.07	17	0.01	19	-1.17	32	-0.24	24	0.00	19	
10	In several aspects, such as the obligations for notification or consultation, the Convention on the Law of the Non-Navigational Uses of International Watercourses fails to abide by the principle of reciprocity, which is a fundamental principle of international law and international relations.	-1.45	35	-0.50	27	-0.35	24	0.00	20	-0.58	26	-0.45	30	0.00	19	
11	The principle of equitable and reasonable utilization in current international water law has not progressed beyond the preliminary levels of simply outlining water quantity allocation and water use division.	-0.76	31	-1.05	31	0.52	10	-1.25	33	0.10	19	-0.32	27	1.68	3	*
12	For projects that may cause significant transboundary harm, countries that share transboundary rivers shall fulfill international obligations to avoid, contain and mitigate such harm.	1.38	5	1.82	2	0.45	12	-0.93	31	0.26	18	0.96	7	0.00	19	**
13	Although "benefit sharing" is frequently mentioned in current international law, this concept lacks a uniform cognition, has not been fully established, and cannot be practically implemented.	0.69	9	-0.21	22	-0.45	26	-1.83	36	0.67	11	1.09	5	-1.88	37	*
14	Water benefit sharing theory requires that water stakeholders to be considered as a whole, with the purpose of promoting the maximization of benefits from the use of transboundary water resources, and emphasizing that all stakeholders can obtain the best benefits.	-0.69	30	1.25	5	-0.22	22	-1.58	34	-0.96	29	1.19	4	-0.78	30	**
15	The core idea of water benefit sharing theory is not to allocate the actual water quantity, but to share the benefits obtained through the development and utilization of water resources.	0.61	12	0.14	16	0.42	14	1.21	5	-0.22	23	-0.80	31	1.88	1	**
16	The ideal water benefit sharing system is a positive-sum game, rather than a zero-sum game where only the amount of water available is allocated.	0.76	7	0.44	11	0.71	7	1.52	3	-0.38	25	-0.10	22	0.12	16	*
17	The benefits of water benefit sharing include environmental benefits to the water resources, such as improvement in water quality, ecological diversity, and environmental sustainability.	0.54	13	0.44	12	-1.42	35	-0.06	22	0.76	8	0.58	11	1.10	6	
18	The benefits of water benefit sharing include direct economic benefits obtained from the uses of water resources, such as hydropower development, agricultural irrigation, and navigation convenience.	0.07	16	0.43	13	-0.19	19	0.67	9	-1.46	36	0.47	12	-0.45	24	
19	The benefits of water benefit sharing include political benefits related to water management, such as the reduction of political costs due to the resolution of international conflicts or the enhancement of international collaboration.	0.07	16	0.73	8	-0.42	25	0.98	7	1.88	2	0.45	13	-0.66	25	*
20	The benefits of water benefit sharing include indirect benefits beyond the water sector, such as promoting infrastructure construction, growth in trade, and so on.	0.61	12	-1.79	36	0.71	6	-0.33	25	2.12	1	-1.39	33	0.98	7	*
21	Among the various benefits gained from water benefit sharing, environmental benefits to the water resources should form the basis of other types of benefits. Environmental benefits will actively promote other types of benefits, while the reverse is not necessarily true.	-0.69	30	-0.44	26	-0.81	29	0.34	13	1.39	3	0.23	18	-0.78	30	*
22	The benefit distribution of the water benefit sharing model could take various forms of equal distribution, proportional distribution according to the required project cost, or equitable distribution of different types of benefits.	-0.61	26	0.13	17	-1.06	32	0.31	15	0.57	12	0.63	9	-0.12	21	
23	Although the principle of benefit sharing does not violate any existing principle of current international water law, it has not become an explicit obligation of any party.	0.61	12	-1.16	33	0.22	16	0.33	14	1.09	5	-0.00	21	1.55	5	**
24	In the context where the principle of good-faith cooperation in international water law applies, countries should at least seriously consider benefit-sharing arrangements proposed by other countries.	0.00	19	0.63	9	-0.22	22	2.20	1	0.68	10	1.47	2	0.66	11	**
25	The idea of water benefit sharing includes the calculation of various aspects and the consideration of relevant benefits. These include for instance, water management in industry, agriculture, domestic uses and ecological protection, which could be understood by the development of a thorough and operable index system of water benefit distribution.	-0.54	25	0.91	6	-1.64	37	-0.01	21	0.05	21	0.32	15	-0.90	31	
26	Due to the lack of any uniform standard or understanding, the concept of water benefit sharing is often ineffective in practice, achieving little more than some hollow "win-win" rhetoric.	-1.84	36	-0.59	28	0.48	11	-1.85	37	0.30	17	-1.71	34	-0.78	30	
27	In the development, use and protection of transboundary river resources, countries that utilize water resources and receive ecological benefits shall correspondingly compensate countries that protect these resources and the ecological environment.	1.38	5	-0.98	29	2.09	2	-0.64	28	1.19	4	-0.43	29	-1.35	35	***
28	In the process of sharing international rivers, ecological benefit compensation should be established as a principle to guide the development, use and protection of transboundary rivers.	-0.22	23	-0.00	20	2.35	1	-0.61	27	0.32	16	0.30	16	1.68	3	**
29	Countries may contribute to the ecological resources of transboundary rivers by taking active measures to protect resources and the ecological environment, such as forestation and the establishment of natural conservation areas.	1.30	6	2.21	1	0.35	15	0.06	17	0.06	20	1.24	3	0.45	13	**
30	Countries may contribute to the ecological resources of transboundary rivers by spontaneously restricting or refraining from certain activities, such as withdrawing from proposed dam construction, halting construction or expansion of industrial or mining operations, or the reduction of forest logging activities.	-0.69	30	1.73	3	1.39	5	1.20	6	0.34	15	0.60	10	-0.78	30	**
31	If the beneficiary countries of ecological resources in transboundary rivers do not provide reasonable compensation to those countries making contributions, it will probably dampen the enthusiasm in protecting transboundary water resources and ecological environment and negatively impact upon the improvement of relations between riparian states.	-1.08	32	-0.21	23	1.58	4	-0.64	29	0.79	7	-0.41	28	0.45	13	*
32	Ecological benefit compensation will address the shortcomings of the existent "polluter pays" principle and stimulate the protection of transboundary rivers and ecological environment.	-0.07	21	0.35	14	-0.26	23	0.34	13	-0.66	27	0.37	14	-1.23	33	
33	Ecological benefit compensation of transboundary rivers ecology may take various forms including financial compensation, material compensation, and policy support.	1.45	3	0.76	7	0.00	18	-0.59	26	0.40	14	1.79	1	-1.10	32	**
34	Under the ecological benefit compensation principle, countries that contribute to the protection of ecological resources and the environment will be motivated by the appropriate compensation they receive, in furthering their protection activities.	0.69	9	1.31	4	0.54	9	0.30	16	0.43	13	0.97	6	-0.08	20	*
35	Under the ecological benefit compensation principles, beneficiary countries that have paid compensation will further appreciate the preciousness of such resources and the ecological environment that they had to pay to preserve.	0.47	14	0.63	10	0.57	8	0.93	8	0.81	6	0.18	20	0.78	10	
36	The ecological benefit compensation principle will facilitate the elevation from individual interest among riparian states to maximizing the collective benefits across the region.	-1.92	37	-0.00	21	1.74	3	-0.90	30	-0.15	22	-0.96	32	-0.21	22	*
37	Any dispute in relation to the ecological benefit compensation principle could be resolved through bilateral or multilateral discussion and negotiation, failing which there may be a judicial recourse to the International Court of Justice.	-0.69	30	-1.05	30	-0.94	31	-0.07	23	0.76	9	0.82	8	0.78	10	

注：①灰色部分代表各因子中的前5位；②"***"表示有三个因子在该命题上的得分排在前三位；"**"表示有两个因子在该命题上的得分排在前三位；"*"有一个因子在该命题上的得分排在前三位。

QSORT		Factor1	Factor 2	Factor 3	Factor 4	Factor 5	Factor 6	Factor 7
China	1	0.0807	**0.7677X**	0.1613	0.1511	0.0174	**0.3842X**	-0.1231
	2	0.3235	0.2545	0.3189	0.2284	0.3262	**0.0449X**	-0.2700
	3	-0.1687	0.2187	0.4939	0.4556	0.1573	**0.0042X**	-0.2982
	4	-0.1986	0.0485	**0.6458X**	-0.1984	-0.1118	**-0.1731X**	0.0008
	5	0.4507	-0.2324	0.3345	-0.3394	-0.0122	**0.1242X**	0.0631
	6	0.0853	-0.1333	-0.0240	-0.3124	**0.5811X**	**0.2482X**	0.0453
	7	0.1316	0.0740	0.1773	**-0.6128X**	0.2175	-0.0181	-0.1340
	8	0.1095	0.1162	**0.7581X**	0.0572	0.1857	-0.0447	0.0853
	9	0.4455	-0.0228	0.3223	-0.0361	-0.4645	-0.0685	0.2307
	10	0.3766	-0.2432	0.3087	0.3993	0.1113	0.1256	0.1006
	11	0.2568	0.3829	0.0112	0.0355	0.0659	-0.2679	0.0281
	12	0.5381	0.0413	0.1268	-0.1921	-0.1674	-0.1347	0.2931
	13	0.2151	-0.0392	0.0018	-0.0009	-0.2275	0.5136	0.0452
	14	0.1995	0.4398	-0.0904	0.3945	-0.0851	-0.5159	0.1770
Southeast Asia	15	0.1334	0.0298	0.2253	**0.6242X**	0.2181	0.1696	0.1393
	16	0.3603	**0.7406X**	0.0187	0.0401	0.0061	-0.2076	0.1408
	17	-0.0106	0.1204	0.1445	-0.1215	0.0831	-0.0400	**0.7978X**
	18	0.0193	**0.5600X**	0.0086	0.3826	-0.1391	-0.0031	0.1368
	19	**0.9048X**	0.0608	-0.0993	0.0291	0.1088	0.1542	0.0494
	20	0.3158	-0.1005	-0.1260	0.0483	0.1803	-0.5469	0.2827
	21	0.1432	**-0.5487X**	-0.2329	0.2014	-0.3439	0.0423	0.1435

	22	-0.0249	0.1019	-0.2174	-0.1147	0.0229	0.1564	0.0785
	23	0.0653	0.0740	-0.0503	-0.0324	**-0.7228X**	0.2372	0.0709
	24	0.0825	-0.0876	0.1259	**-0.6402X**	0.1427	0.0987	0.3851
	25	0.0776	**0.4824X**	-0.4021	-0.1123	0.1828	0.0334	0.0483
	26	0.0924	0.2502	0.1908	0.2022	0.1129	0.1270	0.2183
**India**	27	0.2146	-0.1676	0.2720	0.0870	**0.6142X**	-0.2736	0.0674
	28	-0.1185	**0.6488X**	0.0492	-0.0801	-0.0594	0.0237	-0.1702
	29	0.3268	0.2147	-0.0139	0.0188	**0.6047X**	-0.0326	0.2233
	30	0.0361	-0.2381	-0.0843	0.3523	0.0676	0.0971	**0.7350X**
	31	0.0532	0.0319	**0.5630X**	-0.0535	0.3267	0.1262	0.1210
	32	0.1203	0.1071	-0.1455	0.1425	0.0766	0.7088	0.1198
	33	**0.9048X**	0.0608	-0.0993	0.0291	0.1088	0.1542	0.0494
	34	**0.6683X**	0.1816	0.0454	-0.0779	0.0944	-0.0970	-0.4131
	35	0.0079	0.2264	0.2178	-0.1147	**0.5180X**	0.0977	0.1554
Total		***3***	***6***	***3***	***3***	***5***	***6***	***2***
