# Supplementary material for: Benefit sharing in international rivers: A Q-methodology study of regional understanding and perception in Asia
Source: PLoS One. 2023 Jan 20;18(1):e0280625. doi: 10.1371/journal.pone.0280625 (PMC9857988; doi:10.1371/journal.pone.0280625)
Supplement: Attachment — Submitted filename: Response to Reviewers.docx [file pone.0280625.s002.docx]

Benefit Sharing in International Rivers

Response to reviewers

|  | Comments / Suggestion | Changes Made |
| --- | --- | --- |
| Reviewer 1 | International water law being too narrow, mention UNECE Water Convention | A paragraph added in section 2.1 with specific treatment of 1992 and 1997 Conventions, and them being narrow. |
|  | Limitation of Q-method analysis | Added the constraints and limitation of Q-method analysis in section 3. |
| Reviewer 2 | Keywords should include geographical locations | New keywords added in online submission. |
|  | Definition of ‘benefits’ not clear | Subheading changed to clarify the scope of such ‘benefits’, as these form the substance of this subsection. |
|  | Non-Asia focused paper to be included in the ‘real’ literature review | New non-Asian literature added in section 2.1. |
|  | Limitations should be described | See above for response to Reviewer 1’s suggestion. |
| Journal Requirements | Statement about ethics approval and consent | Statement added in methodology section as well as online submission. |
